# De Quervain Tendinopathy: Anatomical Prognostic Indicators of Corticosteroid Injection Success

**DOI:** 10.3390/jpm14090928

**Published:** 2024-08-31

**Authors:** Dimitrios Kitridis, Evangelos Perdikakis, Michael Potoupnis, Leonidas Pavlidis, Eleni Karagergou, Panagiotis Givissis

**Affiliations:** 1 Faculty of Health Science, School of Medicine, 1st Orthopaedic Department, Aristotle University of Thessaloniki, 54124 Thessaloniki, Greece; ekaragea@auth.gr (E.K.); givissis@med.auth.gr (P.G.); 2 1st Orthopaedic Department, 424 Army General Training Hospital, 56429 Thessaloniki, Greece; 3 Radiology Department, 424 Army General Training Hospital, 56429 Thessaloniki, Greece; e.n.perdikakis@army.gr; 4 Faculty of Health Science, School of Medicine, 3rd Orthopaedic Department, Aristotle University of Thessaloniki, 54124 Thessaloniki, Greece; mikepot@auth.gr; 5 Faculty of Health Science, School of Medicine, Department of Plastic Surgery, Aristotle University of Thessaloniki, 54124 Thessaloniki, Greece; pavlidisl@auth.gr

**Keywords:** de Quervain, tendinopathy, ultrasonography, corticosteroid injection, recurrence

## Abstract

Background: Anatomical variations of the first extensor compartment can affect de Quervain tendinopathy outcomes. Our study aimed to identify the anatomical prognostic indicators of symptom recurrence following a corticosteroid (CS) injection and to assess the efficacy of CS injections. Methods: Fifty consecutive patients received a single CS injection for de Quervain tendinopathy. Ultrasound imaging was used to assess anatomical factors of the first extensor tendon compartment of the wrist. The primary outcome was recurrence after six weeks and six months and the identification of the anatomical prognostic indicators of the recurrence. The Disabilities of the Arm, Shoulder, and Hand (DASH) score and the Visual Analogue Scale (VAS) for pain were evaluated as secondary outcomes. Results: Fifteen patients (30%) experienced symptom recurrence within six weeks. The intracompartmental septum and the number of tendon slips were associated with higher recurrence rates (adjusted odds ratio for the septum: 18.39, *p* = 0.045; adjusted odds ratio for each additional tendon slip: 24.68, *p* < 0.01). The mean DASH score improved from 74.1 ± 5 to 19.3 ± 25.3, and the mean VAS for pain from 8.5 ± 0.8 to 2 ± 2.7 (*p* < 0.01 for both scores). Five patients experienced minor adverse events with spontaneous improvement. Conclusions: CS injections are a viable treatment for de Quervain tendinopathy. Anatomical variations can predict treatment success. Counseling patients based on these factors can help guide treatment decisions, including surgical options.

## 1. Introduction

De Quervain’s disease is the stenosing tenosynovitis of the first extensor compartment of the wrist, affecting the extensor pollicis brevis (EPB) and abductor pollicis longus (APL) tendon sheaths [1]. The tendon sheaths are thickened up to five times their normal size due to myxoid degeneration and fibrous tissue deposition [2,3]. It represents 34% of all tendinopathies of the wrist and hand [4]. Nonsurgical treatments, including bracing and oral anti-inflammatory medications, may be effective for patients with mild symptoms [3,5]. Corticosteroid (CS) injection into the first dorsal compartment may provide durable pain relief even for patients with moderate to severe symptoms [3,6,7,8,9,10]. Surgery is offered when symptoms persist after nonsurgical treatment. 

Several studies demonstrate significant effectiveness of intrasheath CS injections with or without splinting, with success rates ranging from 58 to 93% of cases [3,5,11]. However, the alleviation of symptoms is not universal, and surgical release of the first extensor compartment is necessary in some patients [12]. Studies have mostly correlated the prognosis of CS injections with disease severity and inaccurate technique, without identifying other essential prognostic indicators [6,13]. A limited subset of biological and patient factors that may affect the efficacy of CS injections has been studied, and prior diagnosis of trigger finger or carpal tunnel syndrome was associated with failure of the treatment [6,11,14,15]. Moreover, most surgeons agree that anatomic variations in the tendons and their sheaths, including the abnormal septation of the first extensor compartment contribute both to the course of tendinopathy and to a poor response to conservative treatment [16,17,18,19,20,21,22,23,24,25].

Despite the high rates of effectiveness, extra-articular CS injections may present complications, and the associated risk can be underappreciated. The complications may be major, such as osteomyelitis, necrotizing fasciitis, tendon ruptures, severe soft tissue atrophy, and hypopigmentation, or minor, including minor soft tissue changes, nodules, steroid flare, skin rash, flushing, and menstrual changes [26]. The occurrence of minor soft tissue lesions has been reported in up to 31% of cases due to the superficial location of the first dorsal compartment [26,27,28]. These lesions are commonly transient, however they may occasionally persist [29,30,31]. Despite these complications, most patients prefer that nonsurgical modalities be exhausted first [32].

Recognizing the prognostic factors influencing treatment success could help in counseling patients about the risk/benefit of CS injections. A high possibility of recurrence will determine whether immediate surgical treatment of the disease is indicated, to avoid an unnecessary cycle of conservative treatment along with its potential complications. As previously mentioned, anatomic variations of the first extensor compartment seem to be a factor in both the course of tendinopathy and the recurrence after CS injections. Ultrasound (US) is highly accurate in depicting anatomic variations in the compartment [33,34]. Accordingly, our study aimed to identify the anatomical prognostic indicators of symptom recurrence following a corticosteroid (CS) injection using US imaging and to assess the efficacy of CS injections.

## 2. Material and Methods

### 2.1. Ethical Considerations

We conducted a prospective study of consecutive patients with de Quervain tendinopathy treated with a single CS injection and assessed at standardized follow-up intervals. Formal approval by the institutional review board was obtained a priori (IRB No 6,676/29-07-2020). Written informed consent was obtained from each patient.

### 2.2. Patients and Eligibility Criteria

Consecutive adult patients with a first-time clinical diagnosis of de Quervain tendinopathy presenting at a University Hospital and treated with a CS injection were included. Necessary criteria for the clinical diagnosis were radial-sided wrist pain, tenderness over the first extensor compartment, and positive Finkelstein and Eichhoff tests [1,35]. To perform the Finkelstein test, the thumb was grasped firmly and pulled longitudinally and ulnarly by the examiner, while the other hand held the forearm in a resting position [1]. The Eichhoff’s maneuver, commonly confused with Finkelstein’s test, was performed by having the patient flex the fingers over the involved thumb while the examiner passively ulnarly deviated the wrist [35,36,37]. Both of these maneuvers will cause pain exacerbation. Swelling above the first compartment, while often present, was not considered a required inclusion criterion. These criteria establish the diagnosis of the disease and have been used by other authors as well [6,14]. In cases of bilateral tendinopathy, only the first wrist injected was included to prevent patient bias. Comparing results between wrists could lead to inaccurate patient-reported outcomes. Additionally, anatomical similarities between the wrists could introduce confounding factors.

Exclusion criteria included previous distal radius fracture, inflammatory arthritis, and symptomatic thumb carpometacarpal arthritis with a positive carpometacarpal grind test. Hand and wrist radiographs were obtained to exclude patients with carpometacarpal arthritis or other osseous abnormalities. Patients with tendinopathy in pregnancy or during lactation were also excluded because the disease usually resolves spontaneously [38,39,40].

### 2.3. US Imaging

**Figure 1:**
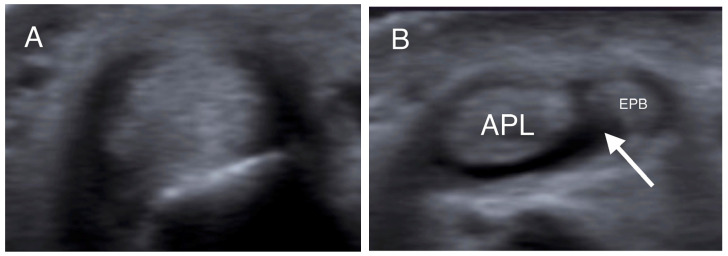
Transverse US images at the level of the distal radius (**A**) and radial styloid (**B**) in the same patient. In (**A**), the tendons could not be individually identified, while in (**B**), the APL and EPB tendons are separated by a hypoechoic septum-like structure (white arrow). (APL: abductor pollicis longus; EPB: extensor pollicis brevis).

**Figure 2:**
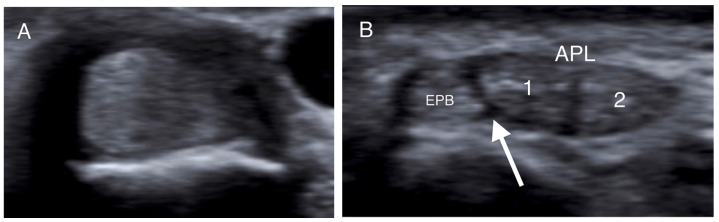

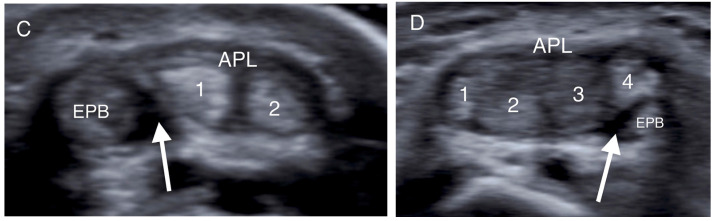
Transverse US images of different patients at the level of the radial styloid. (**A**) The APL and EPB tendons appear as one tendon in the absence of a septum. (**B**,**C**) The intracompartmental septum appears as an intervening hypoechoic area between the APL and EPB tendons (white arrow), while the two separate slips of the APB tendon (1, 2) are identified. (**D**) A hypoechoic septum-like structure (white arrow) separates the APL and EPB tendon slips, suggesting subcompartmentalization; the APL appears with four slips (1–4), and a lotus root sign. (APL: abductor pollicis longus; EPB: extensor pollicis brevis).

**Figure 3:**
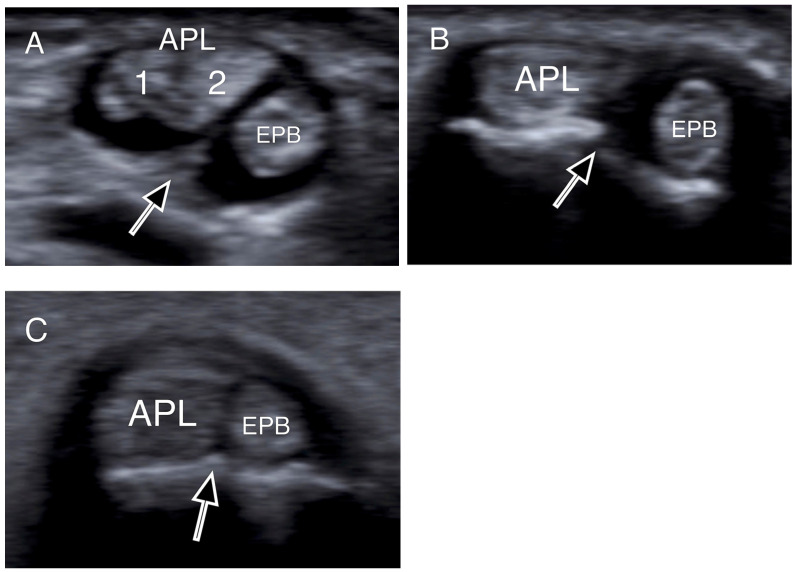
Transverse US images depicting osseous ridges with double groove sign and septums (black arrows). Two APL slips (1, 2) were identified in (**A**), and one slip both in (**B**,**C**). (APL: abductor pollicis longus; EPB: extensor pollicis brevis).

All patients were assessed with US imaging of the first extensor tendon compartment of the wrist. The sonographic evaluations were performed by a senior consultant musculoskeletal radiologist with over 12 years of experience in musculoskeletal imaging (E.P.). All cases were imaged using a US system equipped with a 9–18 MHz linear array transducer and a 9–18 MHz high-resolution linear probe. Tissue harmonics and compound imaging applications were available in specialized musculoskeletal US protocols of the US system. Patients were examined in the sitting position with the affected wrist on a pillow and the radial side of the forearm pointing up. The wrist was examined in the following way: the first extensor compartment was scanned transversely and longitudinally from the point where the first extensor compartment crosses the second compartment to the level of the metacarpal bones (Figure 1). Oblique views were also used to reduce any anisotropy. The APL and EPB tendons were identified laterally and medially respectively. Subcompartmentalization (presence of a septum between APL and EPB) within the first extensor compartment and the number of APL and EPB tendon slips were assessed and recorded (Figure 2). Finally, ultrasonographic signs of intratendinous degeneration (hypoechogenicity, loss of fibrillar echotexture, and intratendinous tears) and the presence of an osseous ridge with a double groove on the bony floor of the compartment were recorded (Figure 3).

More specifically, when the tendons were insonated at right angles to their fibers, presenting as round structures, the intercompartmental septum was observed as an intervening hypoechoic (meaning dark) area between the tendons, running from the extensor retinaculum to the cortex of the radius (Figure 1, Figure 2 and Figure 3) [33,41,42]. The tendons appeared as a single mass when no septum was present (Figure 2). The APL and EPB tendons were identified volarly and dorsally, respectively, and the number of slips of each tendon was assessed and recorded. In the case of multiple slips, the compartment had the appearance of holes in a sliced lotus root, described as a “lotus root sign” (Figure 2) [34].

### 2.4. Injection Technique and Post-Injection Protocol

**Figure 4:**
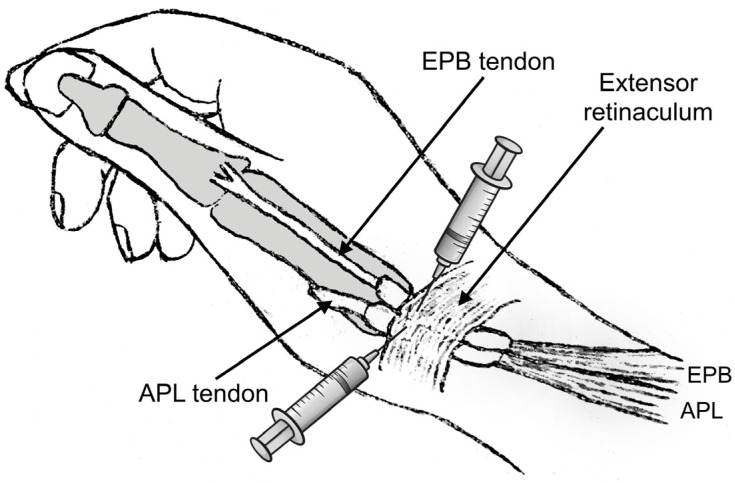
The two-point injection method involves dividing the amount of fluid in half and injecting it into the paths of the abductor pollicis longus (APL) and extensor pollicis brevis (EPB) tendons. The injections should begin at the point of maximum tenderness. (Figure created by the corresponding author).

Although the first extensor compartment was assessed with US imaging in all patients, the CS injections were performed without US guidance to better resemble common clinical practice. The two-point injection technique described by Sawaizumi et al. was used, as it shows superiority compared to the standard one-point technique [43,44]. A solution consisting of 1 mL of betamethasone sodium phosphate—betamethasone acetate mixture and 1 mL of 1% lidocaine was prepared. An experienced hand surgeon provided all the injections (P.G.). The point of maximum tenderness and possible soft-tissue thickening was located and marked with clinical examination. Subsequently, the skin was cleaned with 10% povidone-iodine solution, and after verification of the contours of the APL and EPB tendons, the amount of the injectate was halved and injected into the paths of these two tendons starting from the point of maximum tenderness (Figure 4). Care was taken to avoid injecting directly into the tendon mass or into the subcutaneous tissue to avoid potential rupture or skin depigmentation. Sufficient filling in the tendon sheath distally and proximally was confirmed by palpation.

After the injection, the patients were instructed to wear a removable thumb spica splint for five days and remove it only for bathing. Patients were advised to take acetaminophen 1000 mg in the event of post-injection pain within the first week, as needed for pain management (maximum allowed 3000 mg per day). Upon the completion of the five days of immobilization, patients were encouraged to start using their hand, without following a formal therapy regimen.

### 2.5. Evaluation of Outcomes

The primary outcome was recurrence at either the six-week or six-month follow-up evaluations after the injection, which was defined as the presence of at least one of the initial diagnostic criteria (i.e., presence of radial-sided wrist pain, tenderness to palpation, and positive Finkelstein and/or Eichhoff tests), and the association of the recurrence with the anatomical characteristics of the first extensor compartment of the wrist.

The Disabilities of the Arm, Shoulder, and Hand (DASH) tool and the Visual Analogue Scale (VAS) for pain (where 0 indicated no pain and 10 indicated unbearable pain) were evaluated as secondary outcomes before the injection and at the six-week follow-up [45]. For bilaterally affected patients, the DASH score was focused on the extremity under examination. To interpret the score changes, the minimum clinically important difference (MCID) of −2 for VAS and −14 for DASH score were used [46,47]. 

### 2.6. Statistical Analysis

For the primary outcome, multivariate logistic regression was used to model the association between the recurrence of tendinopathy and independent variables. The existence of intracompartmental septum, the total number of tendon slips of the first extensor compartment, and the existence of a double groove were used as independent variables. Age and gender were inserted in the model to adjust for potential confounding factors. DASH and VAS scores were compared using the Wilcoxon signed ranks test for non-parametric data (the normality of the distributions was assessed with the Kolmogorov-Smirnov test). The level of significance was set at *p* < 0.05 and SPSS software Version 29 was used for all analyses.

The preliminary sample size calculation was performed with the OpenEpi software (OpenEpi.com) [48]. The Kelsey method suggested that with a 0.05 probability of type I error and a power of 80%, a sample size of 42 patients would be necessary to detect an odds ratio of 15 [49,50]. Using the same parameters, the Fleiss method with continuity correction suggested an essential sample of 45 patients [49,50]. A sample of 50 patients was used, accounting for a 10% dropout rate.

## 3. Results

### 3.1. Participants

Fifty consecutive patients with an average age of 50.3 ± 13.4 years participated in the study. There were 10 men and 40 women, and the dominant hand was involved in 30 (60%) cases. Seven patients had bilateral tendinopathy; four had subsequent bilateral injections, but only the first wrist was included in the study. Four patients had hyperthyroidism. The patients’ data are presented in Table 1.

The injections were successfully performed in all cases and there were no withdrawals from the study. In four patients, the pain around the injection site was aggravated; however, the pain markedly improved within the first three days. Skin hypopigmentation was observed in one patient, with spontaneous improvement within the six-month follow-up period. Intracompartmental septum was identified in 27 wrists (54%), and double groove in 12 (24%). The mean tendon slips were two (range, 1–4) for the APL and one (range, 0–2) for the EPB. Signs of intratendinous degeneration were found in all cases.

### 3.2. Primary Outcome

Fifteen patients (30%) had a recurrence of symptoms at the six-week follow-up assessment. Eleven of them subsequently underwent open first compartment release under local anesthesia, and four refused surgery and received a second injection with partial symptomatic relief. The remaining 35 patients (70%) were asymptomatic at both the six-week and six-month follow-up visits. The existence of an intracompartmental septum in the first extensor compartment was predictive of CS injection failure, with increased odds of recurrence (adjusted odds ratio: 18.39, *p* = 0.045). It is worth mentioning that 13 of the 15 cases with injection failure had a septum identified in the compartment. The number of tendon slips was also predictive of symptom recurrence (adjusted odds ratio for each additional tendon slip: 24.68, *p* < 0.01). The presence of double groove, age, and gender were not predictive of injection failure (Table 2). 

### 3.3. Secondary Outcomes

At the six-week follow-up evaluation, 35 patients had complete resolution of symptoms, while the rest had at least partial symptomatic relief after one CS injection. Overall, the injections had a successful outcome; the mean DASH score improved from 74.1 ± 5 to 19.3 ± 25.3, and the mean VAS for pain improved from 8.5 ± 0.8 to 2 ± 2.7, exceeding the MCID. When analyzing the subgroups of patients with recurrence and those free of symptoms, all differences were significant and exceeded the MCID (Table 3).

## 4. Discussion

### 4.1. Efficacy of CS Injections for de Quervain Tendinopathy

In the current study, the success of a single intrasheath CS injection in de Quervain tendinopathy was 70%. The reported success rates vary significantly in the literature, with studies presenting different intervention protocols (i.e., the number and technique of injections) and criteria for successful interventions. A pooled quantitative literature evaluation revealed an 83% cure rate after CS injections [51]. When reporting the results of a single corticosteroid injection, Zingas et al. had a 58% success rate in 19 wrists after three months of follow-up [18]. Likewise, Witt et al. observed 62% relief of symptoms in 87 wrists after a mean of 18 months [52], and Weiss et al. reported 67% in 93 wrists after 13 months; however, 45 of their patients underwent surgery [13]. Earp et al. had an 82% success rate in 50 patients and found that a history of trigger finger reduced the chances of success [14]. 

Better results were reported by other researchers after repeat injections, with Harvey et al. reporting an 82% success rate [27], Anderson et al. 90% [53], and McKenzie et al. 93% [54]. When utilizing US guidance, He et al. reported 73.9% complete resolution of symptoms in a recent systematic review analyzing 10 studies that included 379 wrists [55]. 

### 4.2. Anatomical Prognostic Indicators of CS Injection Success

The exact anatomy of the first dorsal compartment and variations of the first compartment tendons have been thoroughly studied. The first type of variation is the supernumerary tendon slips of the first compartment. Several cadaveric studies found accessory tendons, most commonly of the APL, ranging from 56 to 99% [56,57,58,59,60]. Studies reporting surgical findings on patients with de Quervain’s disease found accessory tendons in 76 to 94% of the patients [61,62,63]. 

Another important variation is the presence of a fibrous septum separating the compartment. Comparative studies have shown that the incidence of septation is increased in patients with de Quervain tendinopathy compared to the general population. Jackson et al. found 40% septation in 300 cadaveric specimens and 67.5% in patients with de Quervain’s [64]. In similar studies, Kulthanan et al. [65] and Aktan et al. [61] observed a significantly increased incidence of septation in de Quervain patients.

Most researchers universally agree that anatomic variations of the tendons of the first extensor compartment and their sheaths contribute to the course of tendinopathy and the poor response to both conservative and surgical treatments [6,16,17,18,19,56,62,63,64,66,67]. Of note, McDermott et al. reported a 14% recurrence rate after US-guided CS injections, all of which had subcompartments on US imaging [11]. However, these anatomical prognostic factors have not yet been studied and quantified in the literature. In the current study, we confirmed that in the case of identification of a septum, the probability of recurrence after one CS injection increases about 18 times (adjusted odds ratio: 18.39, *p* = 0.045), while for any additional tendon slip in the compartment, the probability increases about 25 times (adjusted odds ratio for each additional tendon slip: 24.68, *p* < 0.01).

### 4.3. US Imaging in de Quervain Tendinopathy

Ultrasound (US) is highly accurate in depicting anatomical variations in the compartment. Choi et al. compared the US imaging findings regarding the number of APL and EPB slips and the existence of a septum with surgical records; US imaging presented 100% sensitivity (95% CI: 74–100%) and correctly identified the number of tendon slips in 93% of cases [34]. Kwon et al. in a similar study examined the US identification of the septum and reported 100% sensitivity (95% CI: 80–100%), 96% specificity (95% CI: 78–100%), and 98% accuracy (95% CI: 87–100%) [42]. Nagaoka et al. in another study correctly identified the existence of a septum in 26 of their 27 patients [41]. Rousset et al. in a cadaveric study of 40 specimens reported 95% accuracy in depicting a septum (95% CI: 83–99%) [33]. The accuracies in depicting multiple tendon slips of the APL and EPB were 80% (95% CI: 64%, 91%) and 97% (95% CI: 86%, 100%), respectively [33]. 

### 4.4. Injection and Post-Injection Protocol

The two-point injection technique described by Sawaizumi et al. was used in the current study, as it shows superiority compared to the standard one-point technique [43,44]. The authors evaluated the two techniques in a comparative study of 36 patients and found a significant difference in efficacy favoring the two-point injection group (*p* < 0.001) [43]. In the two-point injection technique, the performer identifies the contours of both the APB and EPL tendons, and the amount of the injectate is halved and injected into both paths to account for subcompartmentalization.

Moreover, the CS injections were performed without US guidance to better resemble common clinical practice. The US-guided injection requires additional equipment in the outpatient clinic and incurs a greater cost to the patient compared to the blind injection, so a high level of clinical evidence is needed for its universal application [68]. The literature currently lacks robust evidence supporting the use of US guidance for CS injections in de Quervain tendinopathy. Two randomized clinical trials have compared blind and US-guided injections. Kume et al. found US-guided injections to be more effective, while Shin et al. reported no difference [68,69]. A recent systematic review by Kathy Shiqi et al. highlights that even with US guidance, symptomatic relief may be limited when a septum is present [70].

There is currently no consensus regarding the use of a splint after the CS injection. Weiss et al., in a retrospective study, compared the use of a thumb spica alone, CS injection alone, and CS injection followed by thumb spica immobilization [13]. Superior outcomes were noted in the CS and the CS with immobilization groups, with no added benefit from the immobilization. Ippolito et al., in another prospective randomized trial, found comparable outcomes when adding immobilization after the CS injection [71]. On the contrary, both Mardani-Kivi et al. [72], in a prospective randomized study, and Cavaleri et al. [73], in a meta-analysis, found the combination of CS injection with immobilization more effective than CS injection alone. In the present study, we applied only five days of thumb spica splinting, shorter than the three weeks utilized in the aforementioned studies, to reduce any post-injection inflammation and discomfort. Then, the patients were instructed to start using their hand without restrictions to avoid the development of kinesiophobia without any added benefit. 

### 4.5. CS Injection Adverse Events

The complications of extra-articular CS injections are usually minor and include minor soft tissue changes, nodules, steroid flare, skin rash, flushing, and menstrual changes [26,74]. The incidence of these complications may be underappreciated; in the case of the first dorsal compartment, the incidence of soft tissue lesions has been reported up to 31%, probably due to the superficial location of the first dorsal compartment [26,27,28]. The lesions are usually transient; however, they may occasionally persist and cause discomfort [29,30,31]. Major adverse events, such as osteomyelitis, necrotizing fasciitis, tendon ruptures, severe soft tissue atrophy, and hypopigmentation, are more rarely encountered [26]. Brinks et al., in a systematic review of 87 studies, reported an incidence of such major adverse events ranging from 0% to 5.8% [74]. In the current study, we noted only minor adverse events, which were transient.

To minimize the incidence of adverse events when injecting CS, the injectate should be aimed and delivered precisely to the target tissues, and the spread to the surrounding soft tissue envelope should be minimized. A moderate needle (23 to 27 gauge) does not create the soft tissue track of a larger needle and is adequate for better aiming and reaching the target than smaller ones [26]. The smallest effective dose of injectate will be the safest, and it should not be injected while withdrawing the needle. The relevant literature reports a variety of dosages and compounds; however, 1 mL of triamcinolone hexacetonide (10 mg/mL) or other CS equivalent doses appear effective for de Quervain tendinopathy [16].

### 4.6. Consulting Patients with De Quervain Tendinopathy

Our findings guide better counseling of patients with de Quervain tendinopathy. After diagnostic US imaging, we now counsel patients that they have about a 70% chance of experiencing relief of symptoms within six weeks after the injection. In the case of the identification of a septum, we also inform them that the probability of recurrence increases about 18 times, while for any additional tendon slip in the compartment, the probability increases about 25 times. Thus, a more personalized approach to treatment is provided, and the patients may elect to proceed with the surgical release of the first extensor compartment if the probability of success of conservative treatment is low. 

### 4.7. Limitations

One limitation of the study was the absence of a control group, which was inherent to the design of our study. Additional potential limitations are the lack of a blinded design and the fact that the same surgeon provided all the injections. Another limitation was that the results of the DASH score for bilaterally affected patients, although focused on the extremity under examination, may be affected by the contralateral tendinitis. Finally, the secondary outcomes of the DASH score and the VAS for pain were evaluated only after six weeks and not at the six-month follow-up assessment. The rationale for assessing the patient-reported outcome scores after six weeks was to evaluate the extent to which patients would experience short-term symptom remission.

Due to the absence of standardized, evidence-based guidelines for injection and post-injection protocols, we employed a common technique without US guidance and provided a short-term splint to all patients. Further high-quality clinical trials are necessary to establish optimal treatment protocols and identify factors influencing individual patient outcomes.

## 5. Conclusions

CS injection is an effective treatment for de Quervain tendinopathy with a 30% recurrence rate and significant improvement in both the DASH score and VAS for pain. The intracompartmental septum and each additional aberrant tendon slip in the first extensor compartment in the US imaging were associated with significantly increased odds of recurrence. Counseling patients based on these factors can help guide treatment decisions, including surgical options. A high risk of recurrence may necessitate immediate surgical intervention to avoid the potential complications of conservative treatment.

## Figures and Tables

**Table 1:** Patients’ baseline characteristics.

Gender (male/female)	10/40
Age (years, mean ± SD)	50.3 ± 13.4
Hand (right/left)	27/23
Dominant hand	30 (60%)
Bilateral involvement	7 (14%)
Intracompartmental septum	27 (54%)
Double groove	12 (24%)
APL tendon slips (mean, range)	2 (1–4)
1 APL slip	17 (34%)
2 APL slips	16 (32%)
3 APL slips	14 (28%)
4 APL slips	3 (6%)
EPB tendon slips (mean, range)	1 (0–2)
No EPB	1 (2%)
1 EPB slip	44 (88%)
2 EPB slips	5 (10%)
Total tendon slips (mean, range)	3 (1–5)

SD: standard deviation; APL: abductor pollicis longus; EPB: extensor pollicis brevis.

**Table 2:** Predictors of recurrence.

Variable	OR_adj_	95% CI	*p*
Septum	18.39	1.07 to 316.16	**0.045**
Tendon slips	24.68	3.22 to 189.26	**0.002**
Double groove	1.47	0.11 to 19.84	0.77
Age	0.99	0.91 to 1.08	0.86
Gender	3.81	0.3 to 47.85	0.3

OR_adj_: adjusted odds ratio; CI: confidence interval. Bold font indicates statistically significant data.

**Table 3:** Pain and functional outcomes.

	Pre-Injection	6 Weeks Post-Injection	*p* ^a^
VAS for pain, all patients (n = 50)	8.5 ± 0.8	2 ± 2.7	<0.01
VAS for pain, no recurrence (n = 35)	8.4 ± 0.8	0.3 ± 0.5	<0.01
VAS for pain, recurrence (n = 15)	8.8 ± 0.8	5.9 ± 1.1	<0.01
DASH score, all patients (n = 50)	74.1 ± 5	19.3 ± 25.3	<0.01
DASH score, no recurrence (n = 35)	73.6 ± 5.4	3.2 ± 4.3	<0.01
DASH score, recurrence (n = 15)	75.4 ± 3.8	56.9 ± 6	<0.01

VAS: Visual Analogue Scale; DASH: Disabilities of the Arm, Shoulder, and Hand score; ^a^: Wilcoxon signed ranks test.

## Data Availability

The original contributions presented in the study are included in the article, further inquiries can be directed to the corresponding author.
